# Microbial Community Affects Daqu Quality and the Production of Ethanol and Flavor Compounds in Baijiu Fermentation

**DOI:** 10.3390/foods12152936

**Published:** 2023-08-02

**Authors:** Pei-Jie Han, Lu-Jun Luo, Ying Han, Liang Song, Pan Zhen, Da-Yong Han, Yu-Hua Wei, Xin Zhou, Zhang Wen, Jun-Zhi Qiu, Feng-Yan Bai

**Affiliations:** 1 College of Life Sciences, Fujian Agriculture and Forestry University, Fuzhou 350002, China; 2 State Key Laboratory of Mycology, Institute of Microbiology, Chinese Academy of Sciences, Beijing 100101, China; luolujun56@163.com (L.-J.L.);; 3 Technology Center, Shanxi Xinghuacun Fen Wine Factory Co., Ltd., Fenyang 032205, China; 4 College of Life Science, University of Chinese Academy of Sciences, Beijing 100049, China

**Keywords:** Daqu of different grades, physicochemical properties, microbial community, PacBio SMRT sequencing, laboratory-scale fermentation trial

## Abstract

Daqu is a traditional starter for Baijiu fermentation and is produced by spontaneous fermentation of ground and moistened barley or wheat. The quality of Daqu is traditionally evaluated based on physicochemical and subjective sensory parameters without microbiological analysis. Here, we compared the physicochemical characteristics of qualified (QD) and inferior (ID) Daqu, their microbial communities based on plate counting and PacBio SMRT sequencing of rRNA gene libraries, and their impacts on Baijiu fermentation. The results showed that the glucoamylase and α-amylase activities of QD were significantly higher than those of ID. The counts of yeasts and relative abundances of functional microbes, especially the amylolytic bacterium *Bacillus licheniformis* and fungi *Saccharomycopsis fibuligera* and *Lichtheimia ramosa*, were significantly higher in QD than in ID. The laboratory-scale Baijiu fermentation tests showed that the relative abundances of the amylolytic microbes were higher in the QD than the ID fermentation set, resulting in more efficient fermentation, as indicated by more weight loss and higher moisture content in the former. Consequently, more glycerol, acetic acid, ethanol, and other volatile compounds were produced in the QD than in the ID fermentation set. The results suggest that Daqu quality is determined by, and can be evaluated based on, its microbial community.

## 1. Introduction

One of the traditional beverages derived from spontaneous fermentation in China is Baijiu, which is a distilled alcoholic product that plays an indispensable role in Chinese culture and the national economy [1]. Baijiu is primarily made from sorghum through a complex process that includes Daqu (or Jiuqu) production and cereal fermentation, distillation, and maturation [2,3,4,5]. Daqu is a traditional, brick-shaped fermentation starter, which is produced in an open environment under non-sterile, semi-controlled conditions via natural microbial inoculation and spontaneous fermentation. The majority of the microbes originate from the raw material and environmental sources, such as tools and the workplace [6,7]. Making Daqu typically comprises three stages: (i) raw material grinding, mixing, and shaping; (ii) artificially temperature-controlled spontaneous solid-state fermentation; and (iii) ripening. The manufacturing processes for Fen Daqu, which is a typical Daqu prepared from barley and pea for the fermentation of light-flavor Baijiu, are shown in Figure S1.

Daqu not only provides the microbial consortium, enzymes (e.g., α-amylase and glucoamylase), and partial raw materials for alcoholic fermentation, but also significantly contributes aroma compounds [2,3,8,9,10,11,12]. Previous studies of Daqu mainly focused on its microbial community profile and enzyme composition [3,4,8,9,13,14,15,16,17,18], the dynamics of microbial succession in the Daqu manufacturing process [19,20,21,22], the effects of fortified Daqu on the microbial composition and flavor ingredients of Baijiu [23,24,25,26,27], and how environmental factors (e.g., moisture, acidity, and temperature) affect the microbial structure and enzymatic activity of Daqu [6,15,28].

Due to the complicated process and multiple factors involved in Daqu production, different grades of Daqu may be obtained. The quality of Daqu is traditionally evaluated based on subjective sensory properties and physicochemical characteristics [29,30,31,32]. A qualified Daqu break usually emits strong and pure ‘Qu-fragrance’, is evenly covered by white spots on over 70% of the surface, and has a relatively thin skin-like structure and a homogenic cross section without obviously visible cracks and mold spots. Moreover, physicochemically, qualified Daqu (QD) usually has higher glucoamylase and α-amylase activities than inferior Daqu (ID). Previous studies provided a preliminary understanding of the microbial community and volatile compounds in Daqu of normal and better grades. Researchers compared the bacterial communities of different grades of roasted-sesame-like flavored Daqu, but did not reveal the link between flavor compounds and the bacterial communities [29]. Zhang et al. (2022) demonstrated that superior- and normal-grade sauce-flavor Daqu had distinct flavors. In addition, fungal counts were higher in normal-grade Daqu than superior-grade Daqu, while bacterial community richness was higher in superior-grade Daqu than in normal-grade Daqu [32]. However, there have been few comparative studies of the microbial community and physicochemical properties of QD and ID and the effects of different quality levels of Daqu on the end products of fermentation.

To address this gap, the physicochemical features of qualified and inferior Fen Daqu (light-flavor Daqu) were compared in the present study. Culture-dependent and PacBio small-molecule real-time (SMRT) sequencing methods were used to obtain an overview of the microbial composition of QD and ID. The effects of QD and ID on the fermentation parameters and on ethanol and flavor-related compound production were further elucidated by simulating the Baijiu fermentation process at laboratory scale. Our findings shed new light on the microbiological and physicochemical differences of Daqu with different qualities and the impact of these differences on the end products, thus providing theoretical support for evaluating Daqu quality and manipulating the Daqu manufacturing process to improve Baijiu quality.

## 2. Materials and Methods 

### 2.1. Sample Collection

The traditional manufacturing processes for Fen Daqu and Baijiu are schematically depicted in Figure S1. A total of 25 brick-shaped Fen Daqu samples, including 17 qualified (coded as QD1-QD17) and 8 inferior (coded as ID1-ID8) samples, were collected from Shanxi Xinghuacun Fenjiu Distillery Co., Ltd. (Fenyang, China) in August 2021. The qualities of the Daqu samples were determined according to Daqu quality evaluation principles by experts of Daqu manufacture in the distillery. Each sample was ground to fine power and divided into two portions. One portion was used for physicochemical, microbiological, and laboratory-scale fermentation analyses, and the other was used for DNA extraction.

### 2.2. Physicochemical Properties

The moisture content, pH, total titratable acidity, and glucoamylase and α-amylase activities of each Daqu sample were determined in three independent analyses. After drying at 105 °C for 6 h, the moisture content of the Daqu powder was calculated using the gravimetric method [33]. pH was determined with a pH Meter AS700 (AS ONE Corporation company, Osaka, Japan). Total titratable acidity was determined by titration with 0.1 M NaOH and 100 μL of phenolphthalein to obtain a titration endpoint of pH 8.2. Glucoamylase and α-amylase activities were determined as described previously [28].

### 2.3. Isolation and Enumeration of Different Culturable Microbes

Twenty grams of Daqu powder were homogenized in 180 mL of sterile 20 mM phosphate-buffered saline (PBS), pH 7.5, and incubated for 20 min at 150 rpm and 25 °C. This suspension was defined as a 1/10 dilution and was serially diluted 10-fold with PBS. A 100 μL aliquot of each dilution was spread and incubated on the following agar media: (i) yeast extract peptone dextrose agar (YPD) supplemented with 3.3 mL/L acetic acid (YPD-AA agar) [34] and incubated at 30 °C for 72 h for enumeration of yeasts; (ii) 25% glycerol nitrate agar (G25N) incubating at 30 °C for 120 h to selectively isolate and enumerate *Saccharomycopsis fibuligera* [35]; (iii) Rose Bengal agar (RBA) incubating at 37 °C for 48 h to enumerate molds; (iv) potato dextrose agar (PDA) incubating at 45 °C for 72 h to enumerate thermophilic or thermotolerant molds; (v) Reasoner’s 2A agar (R2A) incubating at 37 °C for 48 h to enumerate aerobic bacteria; (vi) de Man, Rogosa, and Sharpe agar (MRS) incubating at 37 °C for 72 h to enumerate lactic acid bacteria (LAB); and (vii) beef extract peptone agar (RZ) incubating at 37 °C for 72 h to enumerate *Bacillus*. For (vii), the suspension was heated at 75 °C for 15 min prior to plating.

The media for culturing fungi and bacteria were supplemented with final concentrations of 100 g/L chloramphenicol and 8 g/L amphotericin B, respectively. The enumerations of each group of microbes for each sample were performed in triplicate. The above reagents were purchased from Solarbio Science & Technology Co., Ltd., Beijing, China.

### 2.4. Genomic DNA Extraction 

Twenty grams of Daqu powder was homogenized in 180 mL of sterile distilled H_2_O and incubated at 20 °C for 20 min at 150 rpm, and 4 mL of the suspension was subsequently centrifuged at 12,000 rpm (the same speed was used in the following steps) for 6 min at 4 °C. Genomic DNA was extracted from the pellet according to a previously reported procedure with some modifications [36,37]. The details of the procedures were described previously [38].

### 2.5. DNA Amplification 

The primer pair ITS1F (5′-CTT GGT CAT TTA GAG GAA GTA A-3′) and ITS4 (5′-TCC TCC GCT TAT TGA TAT GC-3′) was used to amplify the entire ITS region of fungi [39]. The primers were used from the previous study to amplify the 16S rRNA genes of bacteria [40]. Each primer contained an 8-nucleotide barcode label as a multiplexing marker. Each 25 μL polymerase chain reaction (PCR) contained 1 μL of 50 μg/mL genomic DNA, 12.5 μL of KOD Mix (Catalog Number: KMM-101NV, manufactured by Toyobo and distributed by Sigma-Aldrich, Merck KGaA^TM^, Darmstadt, Germany), 1 μL of each 0.1 M primer, and 9.5 μL of nuclease-free H_2_O. The 16S rRNA gene was amplified using the following conditions: denaturation at 98 °C for 2 min, 32 cycles of denaturation at 98 °C for 10 s, annealing at 55 °C for 10 s, extension at 72 °C for 22 s, and a final extension at 72 °C for 5 min. The ITS amplification procedure was 98 °C for 2 min, 32 cycles of denaturation at 98 °C for 10 s, annealing at 56 °C for 10 s, extension at 72 °C for 15 s, and a final extension at 72 °C for 5 min. SMRTbell libraries (Pacific Biosciences of California, Inc., San Francisco, CA, USA) were constructed according to the manufacturer’s instructions. Sequencing was performed by the Biomarker Technologies Corporation (Beijing, China). 

### 2.6. Sequence Data Processing and Statistical Analysis

The methods of the sequence data processing were reported previously [38,41,42,43]. To analyze the diversity of bacteria and fungi, R version 4.1 (R Core Team) and R packages dplyr, pheatmap, igraph [44], and vegan [45] were used. The richness index was determined using the vegan functions ‘diversity’ and ‘estimateR’ [45]. Differences in alpha diversity and taxonomy between groups were analyzed by independent one-way analysis of variance (ANOVA) followed by Tukey’s honestly significant difference test (*p* < 0.05). Beta diversity was analyzed using Bray–Curtis dissimilarity distances and visualized via principal coordinate analysis (PCoA) plots [45].

The negative binomial generalized linear model was used to calculate significantly enriched bacterial or fungal ASVs in QD and ID using the edgeR package in R [46]. All data were first normalized using the trimmed mean of M-values (TMM) method, and the final *p* values were corrected using the false discovery rate (FDR) method. Only ASVs with FDR < 0.05 were retained for the visualization of significantly enriched ASVs in volcano plots. The microbial communities of QD and ID were further compared using the linear discriminant analysis effect size (LEfSe) analysis with the online interface using the Huttenhower Lab Galaxy Server: http://huttenhower.sph. harvard.edu/galaxy/ (accessed on 13 May 2023).

Co-occurrence networks of the bacterial and fungal communities were constructed based on high-abundance ASVs in the Daqu and fermentation groups. Significant and robust Spearman rank correlations (correlation values < −0.7 or > 0.7 and *p* < 0.05) were calculated using the igraph package in R [44]. Only significant correlations between different microbial ASVs were used to construct co-occurrence networks. Next, the occurrence network modules in which the nodal substructure had a higher density of edges within groups than between groups were calculated using the “greedy optimization of modularity” algorithm. Separate co-occurrence networks were constructed and visualized according to the different compartments of the network topological characteristics using the Fruchterman–Reingold layout algorithm (10^4^ permutations). The topological characteristics included the numbers of edges, modules, and nodes, as well as the density and modularity, which represent the complexity and connectivity of the co-occurrence network, respectively.

### 2.7. Laboratory-scale Fermentation

To investigate the effects of Daqu quality on the yield and flavor of Baiju, we performed laboratory-scale solid-state fermentations using QD and ID as starters. The fermentation process mimicked the process of traditional light-flavor Baiju production in the distillery. Firstly, 4 kg of crushed sorghum was mixed with 2.8 kg of 90 °C water and soaked at room temperature for 20 h. The steeped sorghum was steamed for 1 h, and an aliquot of 280 g of the steamed sorghum was cooled to 16 °C and mixed thoroughly with 15 g of Daqu. Then, the mixture was put into a laboratory-scale bioreactor (Figure S2a). Four QD and four ID samples were randomly selected and used in the fermentation experiment and three duplicates were set for each sample. Three bioreactors containing soaked sorghum without Daqu were employed as a control (CK) set. The fermentation period was 28 days, and the temperature was adjusted every 24 h to mimic the ideal traditional fermentation temperature curve (Figure S2d). At the beginning and end of fermentation, the fermentation mass was weighed for weight loss calculation. All analyses were performed in triplicate. For each fermentation repeat, an aliquot of 20 grams was sampled at the beginning and the end of fermentation and DNA was extracted from the sample using the same method as described above for DNA extraction from Daqu.

### 2.8. Flavor Compounds Analysis 

High-performance liquid chromatography (HPLC) was performed as described previously with minor modifications [47]. Briefly, 5 g samples were added to 45 mL of distilled H_2_O, and the mixture was placed in ice water in an ultrasonic tank for 30 min and centrifuged at 3500 rpm for 20 min. The supernatant was passed through a 0.22 μm syringe filter and quantified by HPLC on a system equipped with a refractive index detector (RID-20A, Shimadzu, Kyoto, Japan) and an Aminex HPX-87H column (300 mm × 7.8 mm, Bio-Rad Laboratories Inc., Contra Costa, CA, USA). The column temperature was 30 °C. The mobile phase was 5 mM H_2_SO_4_ at a flow rate of 0.3 mL/min. The sample injection volume was 10 μL. The concentrations of acetic acid, lactic acid, glycerol, and ethanol were calculated from the peak areas based on standard samples.

The volatile compounds were performed by headspace solid phase microextraction and gas chromatography–mass spectrometry (HS-SPME-GC-MS) according to the protocol described previously [47].

## 3. Results 

### 3.1. Physicochemical Characteristics of Qualified and Inferior Daqu

**Figure 1:**
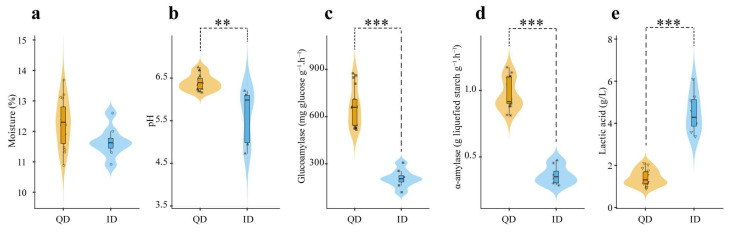
Physicochemical characteristics of qualified Daqu (QD) and inferior Daqu (ID). (**a**) Moisture; (**b**) pH; (**c**) glucoamylase activity; (**d**) α-amylase activity; (**e**) content of lactic acid. The values were from 17 QD and 8 ID samples. **, 0.001 ≤ *p* < 0.01; ***, *p* < 0.001.

The physicochemical properties including moisture, pH, and glucoamylase and α-amylase activities used as key criteria for evaluating Daqu quality in the distillery were measured (Figure 1). There was no significant difference in moisture content between QD and ID. However, the average pH of ID (5.61) was significantly lower (*p* < 0.05) than that of QD (6.39). The average lactic acid content of ID (4.49 g/L) was significantly higher (*p* < 0.05) than that of QD (1.42 g/L). The glucoamylase and α-amylase activities of QD were 667.76 mg glucose g^−1^ h^−1^ and 0.97 g liquefied starch g^−1^ h^−1^, respectively, and were 281% and 269% higher than those of ID, respectively. 

### 3.2. Enumeration of Microorganisms in Daqu

**Figure 2:**
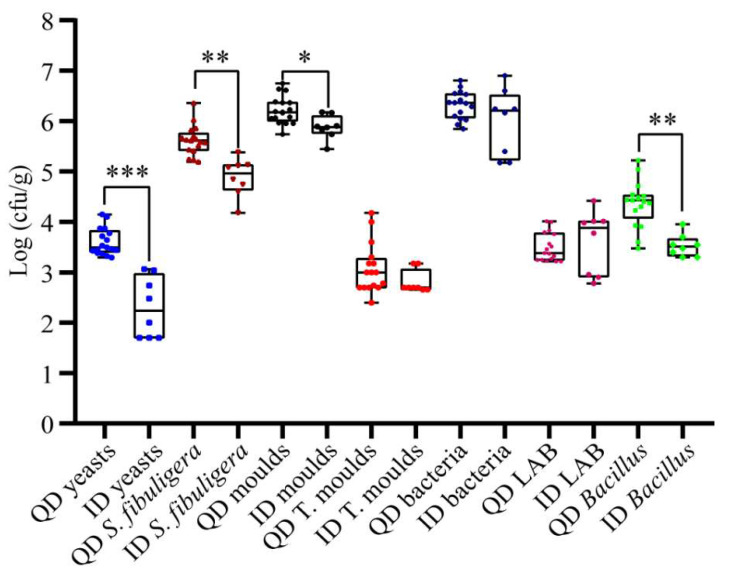
Microbial enumerations of qualified Daqu (QD) and inferior Daqu (ID) samples. *S. fibuligera*, *Saccharomycopsis fibuligera*; T. moulds, thermophilic or thermotolerant molds; LAB, lactic acid bacteria. Data from 17 QD and 8 ID samples are shown. *, 0.01 ≤ *p* < 0.05; **, 0.001 ≤ *p* < 0.01; ***, *p* < 0.001.

Yeasts, filamentous fungi, and bacteria were enumerated by culture-dependent plating analysis. As shown in Figure 2, on average, the yeast count was significantly higher in QD than in ID (3.61 log cfu/g vs. 2.29 log cfu/g, *p* < 0.001). ID had significantly lower (*p* < 0.05) average counts of *Saccharomycopsis fibuligera*, molds, and mesophilic *Bacillus*, with values of 4.88, 5.87, and 3.53 log cfu/g, respectively. However, the total enumeration of aerobic bacteria and LAB did not differ significantly between QD and ID (*p* > 0.05). 

### 3.3. Microbial Landscape Revealed by PacBio SMRT Sequencing

Genomic DNA was extracted from each Daqu sample and from laboratory-scale fermentations for PacBio SMRT sequencing. After filtering the raw sequencing data, a total of 625,410 and 582,863 high-quality PacBio SMRT sequencing reads were obtained for bacterial 16S and fungal ITS rDNA, respectively. On average, 7916 bacterial and 7378 fungal reads were obtained for each sample. The bacterial and fungal reads were assembled into 5876 and 156 OTUs, respectively. The rarefaction curves of different sample groups are shown in Figure S3. The sequences belonged to 6 phyla, 63 genera, and 161 species of bacteria and 3 phyla, 26 genera, and 39 species of fungi.

**Figure 3:**
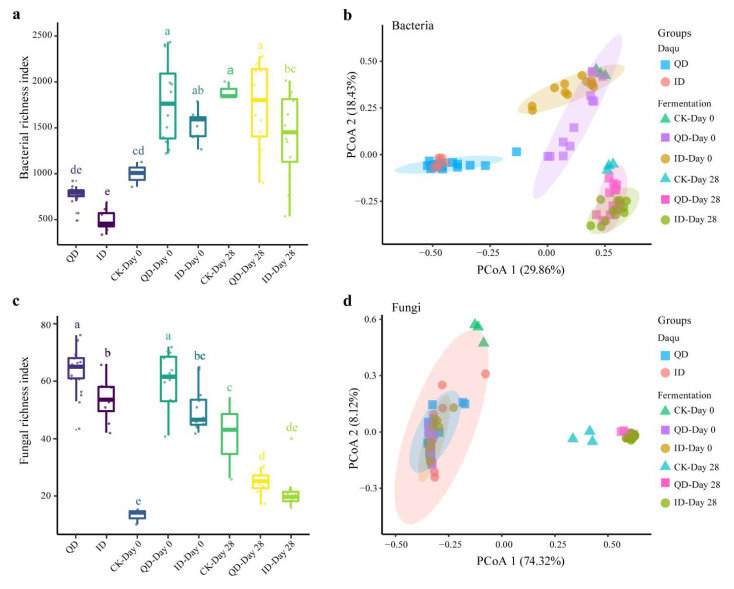
Microbial richness and β-diversity in qualified (QD) and inferior (ID) Daqu samples and in the samples at the beginning (Day 0) and the end (Day 28) of the laboratory-scale Baijiu fermentation tests. (**a**) Bacterial and (**c**) fungal richness index. Principal coordinate analysis (PCoA) plots of bacterial (**b**) and fungal (**d**) sample groups, which were created based on the Bray–Curtis dissimilarity distance. CK, control group. Bars with different letters in a and c indicate significant difference (*p* < 0.05).

Comparative bacterial and fungal diversity analyses were carried out after normalizing the sequence numbers to ensure equal sequencing depths. The bacterial diversity was significantly higher in the QD samples than in the ID samples and in the fermentations inoculated with QD than in the fermentations inoculated with ID (Figure 3a). The PCoA plot revealed that the bacterial community composition differed significantly between the QD and ID groups (Figure 3b). The fungal community diversity was also significantly higher in QD than in ID (Figure 3c). In both QD and ID fermentations, the fungal community diversity decreased significantly during fermentation. The fungal communities in the fermentation tests without Daqu inoculation (the control groups) were clearly separated from the others, indicating the significant effect of Daqu inoculation (Figure 3d). The ANOSIM test revealed significant differences (*p* < 0.05) in the bacterial communities between the QD and ID samples and between the QD and ID fermentation tests at the beginning (Day 0) and the end (Day 28) stages, respectively (Table S1). The initial fungal communities (Day 0) in QD and ID fermentation tests also differed significantly (Table S1).

#### 3.3.1. Microbial Community Composition and Co-occurrence Networks of QD and ID

**Figure 4:**
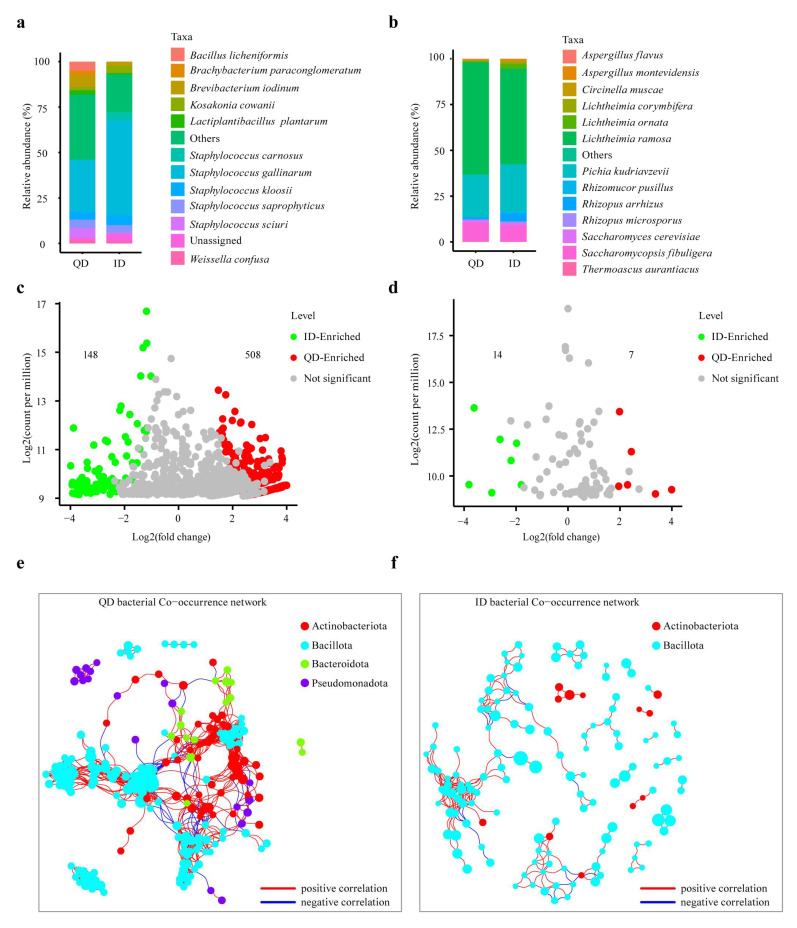
Microbial community compositions, specificities, and co-occurrence networks in qualified (QD) and inferior (ID) Daqu. Others, the sum of the taxa with a relative abundance < 0.9%; unassigned, those that did not match any known species. (**a**) Bacterial and (**b**) fungal community composition at the species level. Volcano plots of bacteria (**c**) and fungi (**d**) were created based on amplicon sequence variants’ (ASVs) fold change and counts per million. Each dot represents an ASV, the red dots represent the ASVs that are significantly enriched in QD (FDR < 0.05), the green dots display the ASVs that are significantly enriched in ID (FDR < 0.05), and grey dots show ASVs that are not statistically different between QD and ID. Bacterial co-occurrence networks of QD (**e**) and ID (**f**) were constructed based on ASVs. Nodes represent ASVs and are colored by phylum, and links between the nodes show significant correlations.

Twelve bacterial genera at an average relative abundance > 1% were found in the 25 Daqu samples compared (Figure S4a, Table S2). The average relative abundance of the dominant family Staphylococcaceae was 77.6% in ID, 1.74 times higher than that in QD (Figure S4b). The top 10 genera were *Staphylococcus*, *Brevibacterium*, *Lactobacillus*, *Weissella*, *Bacillus*, *Brachybacterium*, *Sphingobacterium*, *Lactiplantibacillus*, *Corynebacterium*, and *Kocuria*. The bacterial genera and species in QD and ID were largely similar, but their proportions were different. In QD, *Staphylococcus gallinarum* (28.4%), *Brevibacterium iodinum* (6.4%), *Staphylococcus sciuri* (5.2%), *Bacillus licheniformis* (5.0%), *Staphylococcus saprophyticus* (4.7%), and *Staphylococcus kloosii* (4.5%) were the top six species (Figure 4a), with a total proportion of 54.1%. In ID, the top six species were *Staphylococcus gallinarum* (52.2%), *Staphylococcus kloosii* (5.3%), *Staphylococcus carnosus* (4.7%), *Staphylococcus saprophyticus* (4.1%), *Kosakonia cowanii* (4.1%), and *Brevibacterium iodinum* (2.0%).

The fungal communities in QD and ID were less diverse than the bacterial communities. Six fungal genera with a relative abundance > 1% were identified from the Daqu samples (Table S2, Figure S4c). The most dominant family was Lichtheimiaceae, which occupied a relative abundance of above 50% in both QD and ID (Figure S4d). The top six fungal genera were *Lichtheimia*, *Pichia*, *Saccharomycopsis*, *Rhizopus*, *Saccharomyces*, and *Aspergillus*. In QD, the top six species, which represented 98% of the total abundance, were *Lichtheimia ramosa* (61.2%), *Pichia kudriavzevii* (23.0%), *Saccharomycopsis fibuligera* (10.3%), *Rhizopus arrhizus* (1.4%), *Saccharomyces cerevisiae* (1.5%), and *Lichtheimia ornate* (0.6%). In ID, the top six species, which represented 95.7% of the total abundance, were *Lichtheimia ramosa* (52.1%), *Pichia kudriavzevii* (25.8%), *Saccharomycopsis fibuligera* (9.1%), *Rhizopus arrhizus* (4.5%), *Lichtheimia ornata* (2.7%), and *Lichtheimia corymbifera* (1.4%) (Figure 4b).

The volcano plots indicated that 508 and 148 bacterial ASVs were highly enriched in QD and ID (Figure 4c), respectively, while 7 and 14 fungal ASVs were highly enriched in QD and ID, respectively (Figure 4d). The bacterial co-occurrence networks differed significantly between QD and ID (Figure 4e,f). The bacterial co-occurrence network of QD was much more complicated, with a longer average path length, more nodes and edges, greater network density, and higher modularity (Figure 4e and Table S3). Similar differences were observed in the fungal co-occurrence networks between QD and ID (Figure S4e,f). These findings suggested that the microbiome networks of QD facilitated more interactions between diverse species and were more stable than the ID microbiome networks.

LEfSe analysis showed that a total of 27 bacterial and two fungal taxa were significantly different between QD and ID in terms of abundance (Figure S5). *Staphylococcus* was the most significantly enriched bacterial genus in ID (Figure S5b), while *Thermoascus* was the most significantly enriched fungal genus in QD (Figure S5d).

#### 3.3.2. Microbial Composition during Fermentation

**Figure 5:**
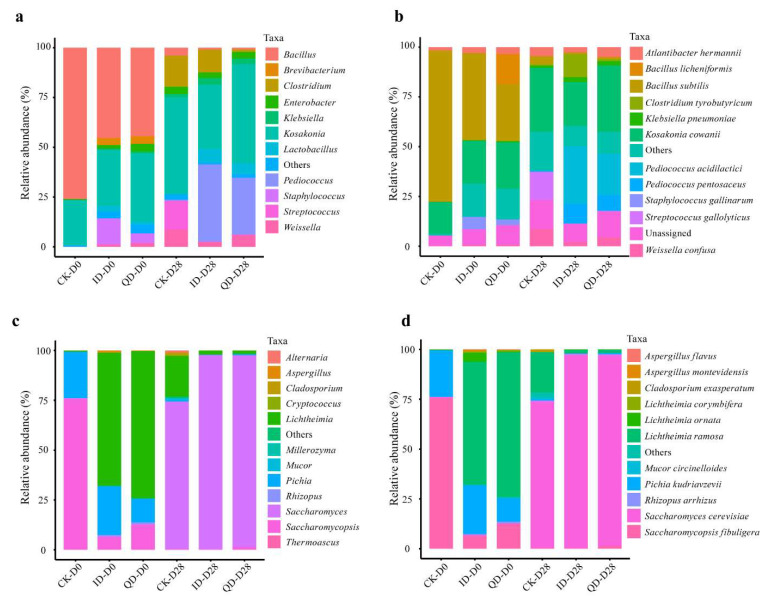
Relative abundances of bacteria (**a**,**b**) and fungi (**c**,**d**) in the laboratory-scale fermentations at the species and genus levels.

The initial relative abundances of some bacteria and fungi differed significantly between the QD and ID fermentations. At the genus level, the bacterial genus *Staphylococcus* was much more abundant in the ID set (13.1%) than in the QD set (4.7%) (Figure 5a, Table S4). This genus was also identified as a significant discriminant taxon of the initial bacteria community in the ID fermentation group (Figure S6b). At the species level, the proportion of *Bacillus licheniformis* in the QD set (15.3%) was 76 times higher than in the ID set (0.2%), whereas the proportions of *Bacillus subtilis* and *Staphylococcus gallinarum* in ID (43.6% and 6.2%, respectively) were 1.5 and 2.2 times higher than in the QD set (28.5% and 2.8%, respectively) (Figure 5b, Table S4). Among the fungi, *Lichtheimia*, *Saccharomycopsis*, *Rhizopus*, and *Hyphopichia* were the most significantly enriched genera at the beginning (Day 0) for the QD fermentation group (Figure S6d). The initial percentages of *Lichtheimia ramosa* and *Saccharomycopsis fibuligera* were 72.7% and 12.4% in QD fermentation but 61.7% and 6.26% in ID fermentation, respectively (Figure 5c,d; Table S4).

At the end of fermentation, the bacterial genera and species with a relative abundance > 1.0% were similar in the QD and ID fermentations (Figure 5a,b). However, their proportions varied; for example, the proportions of *Pediococcus acidilactici* and *Clostridium tyrobutyricum* were 28.9% and 11.5%, respectively, in the ID fermentation, but 20.8% and 1.1% in QD fermentation (*p* < 0.05) (Figure 5b, Table S4). LEfSe analysis indicated that *Clostridia* was a discriminant bacterial taxon in the final microbial communities of the ID fermentation set (Figure S6b). The dominant fungal genus and species were shifted to *Saccharomyces* and *Saccharomyces cerevisiae*, which accounted for more than 95% of the fungal community in both QD and ID fermentations (Figure 5c,d).

### 3.4. Physicochemical Characteristics and Metabolic Profiles of the Fermentation End Products

**Figure 6:**
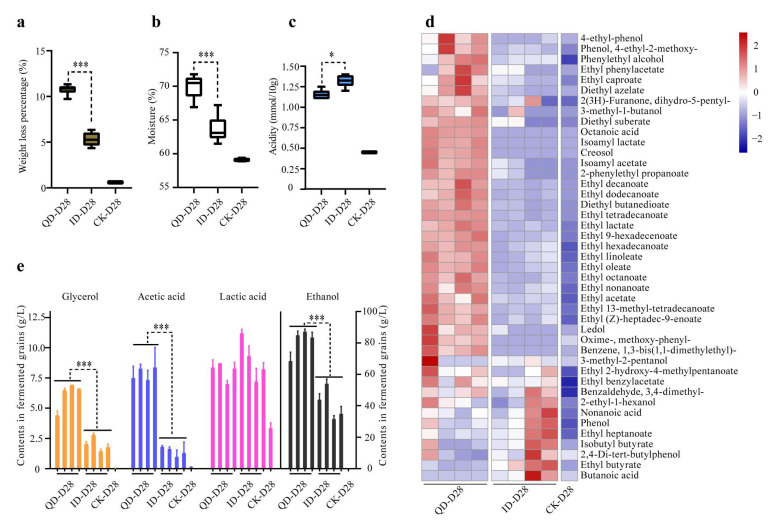
Physicochemical properties and flavor compounds of the end products in the laboratory-scale fermentation tests using qualified (QD) and inferior (ID) Daqu as starters. (**a**) Weight loss percentage; (**b**) moisture; (**c**) titratable acidity; and (**d**,**e**) flavor compounds. The volatile compounds in the heatmap were transformed by z-score in (**d**). *, 0.01 ≤ *p* < 0.05; ***, *p* < 0.001. Error bars, standard deviations (*n* = 3). CK, control.

To study the effects of QD and ID on the end products of fermentation, we determined physicochemical properties at the end of fermentation, including weight loss, moisture content, and total titratable acidity (Figure 6). On average, the weight loss rate in the QD fermentations (10.7%) was significantly higher (*p* < 0.001) than that in the ID fermentations (5.4%) (Figure 6a). The average moisture contents of the end products of the QD and ID fermentations were 70.0% and 63.7%, respectively (Figure 6b). The end products of the ID fermentations had significantly higher total titratable acidity than the end products of the QD fermentations (*p* < 0.05) (Figure 6c).

The metabolic profiles of the end products of fermentation were determined using HS-SPME-GC-MS and HPLC analyses. The HS-SPME-GC-MS analysis identified a total of 43 volatile metabolites, including three acids, five alcohols, six aromatic compounds, 26 esters, one heterocyclic compound, one carbonyl compound, and one nitrogenous compound (Figure 6d). The concentrations of the majority of the volatile compounds, including ethyl linoleate, ethyl oleate, ethyl hexadecanoate, ethyl hexanoate, ethyl acetate, ethyl lactate, and phenylethyl alcohol, were significantly higher in the QD than in the ID fermentation products. In contrast, the concentrations of ethyl butyrate and butanoic acid were significantly higher in the ID than in the QD fermentation products (*p* < 0.001) (Figure 6d).

The HPLC results showed that the QD fermentation products contained 6.1 g/L glycerol, 7.8 g/L acetic acid, and 80.8 g/L ethanol on average. These metabolites were significantly less (*p* < 0.001) in the ID fermentation products, which contained 2.0 g/L glycerol, 1.4 g/L acetic acid, and 40.8 g/L ethanol on average. The lactic acid concentrations were not significantly different (*p* > 0.05) between the QD and ID fermentations (Figure 6e).

## 4. Discussion

The present study compared the physicochemical and microbial properties of Fen Daqu samples of different quality and analyzed the effects of Daqu quality on the end products of Baijiu fermentation. We found significant differences between QD and ID in certain key physicochemical characters and in their microbial communities. The results suggest that the quality of Daqu is mainly determined by its microbial community, which contributes the majority of the microbes involved in the fermentation of Baijiu and thus significantly affects ethanol yield and the generation of flavor compounds in the end product of the fermentation.

The quality of Daqu is traditionally assessed based on subjective sensory evaluation and physicochemical analysis [29,30]. For a Fen Daqu brick, the sensory evaluation parameters include the coverage of white spots on the surface, the thickness of a skin-like structure, the texture of the cross section, and smell [31]. The physicochemical criteria include moisture content and the starch degradation and saccharification abilities. The microbial index has not been considered in the quality evaluation of Daqu, even though Daqu is prepared by spontaneous fermentation for the enrichment of the microbes responsible for Baijiu fermentation and the parameters used in the traditional evaluation mostly result from microbial activities in the Daqu. Specifically, the white spots on the surface of a Daqu brick are mainly formed by *Saccharomycopsis fibuligera*, which is an amylolytic yeast that plays a key role in the starch saccharification and ester and alcohol compound production in Baijiu fermentation [48,49]. The smell is mainly contributed by the metabolites of microbes involved in Daqu fermentation. The starch degradation and saccharification abilities are assessed based on glucoamylase and α-amylase activities, which are usually contributed by amylolytic microbes, including bacterial species such as *Bacillus licheniformis* and fungal species in the genera *Lichtheimia*, *Rhizopus*, *Aspergillus*, and others, in addition to *Saccharomycopsis fibuligera*. The results of this study suggest that the microbial community can be considered as an indicator of Daqu quality.

In order to compare the difference in the count of the amylolytic yeast *Saccharomycopsis fibuligera* between QD and ID, we tried different media and found that G25N agar is the best medium for isolating and counting this species. The colonies of *Saccharomycopsis fibuligera* growing on G25N agar can be easily distinguished from those of other yeasts and molds. Our results showed that QD contained significantly more *Saccharomycopsis fibuligera* than ID (Figure 2), being consistent with the criterion that the surface of a QD brick is covered by more white spots than that of an ID brick. In addition to *Saccharomycopsis fibuligera*, other yeasts are important for ethanol and flavor compound production during Baijiu fermentation. A previous study of ours showed that the common YPD agar supplemented with 3.3 mL/L acetic acid (YPD-AA) is suitable for selective isolation and counting of yeasts, because the growth of filamentous fungi was usually inhibited and yeasts generally grow well on this medium [34]. In this study, we showed that the counts of yeasts on YPD-AA were significantly higher in QD than in ID (Figure 2). The results suggest that the counts of *Saccharomycopsis fibuligera* on G25N agar and of other yeasts on YPD-AA ager can be used to evaluate the quality of Fen Daqu.

The difference in microbial communities between QD and ID was further revealed by culture-independent analysis based on PacBio SMRT sequencing. Illumina sequencing generates shorter sequence reads and thus usually results in low taxonomical resolution to only the genus level [14]. In contrast, PacBio SMRT sequencing is a high-speed and more sensitive method that generates much longer sequence reads, thereby improving the accuracy of the molecular identification of microbes and resulting in relatively high taxonomic resolution to the species level of the microbial community in a specific ecosystem [50,51], though with a relatively higher cost and lower throughput. Previous studies on the microbial diversity of Daqu using culture-independent methods, usually based on high-throughput Illumina sequencing of DNA libraries, consisted of amplicons of partial 16S (usually the V3-V4 region for bacteria) or partial ITS (usually the ITS1 or ITS2 region for fungi) rDNA, and thus the bacteria and fungi detected were only identified to the genus level [52]. The present study sequenced the full 16S and ITS rDNA units and thus the bacteria and fungi in the Daqu samples that were compared were more accurately identified to the species level. We found that the species diversities of both bacteria and fungi were significantly higher in QD than in ID (Figure 3).

Though the dominant microbial species harbored in QD and ID were largely similar (Figure 4), the proportions of individual species were significantly different. Notably, among the dominant bacterial species, the proportions of *Bacillus licheniformis*, *Lactiplantibacillus plantarum*, and *Staphylococcus sciuri* were remarkably higher in QD than in ID (Figure 4a). *Bacillus licheniformis* is an important functional species in Baijiu fermentation which produces carbohydrase and protease enzymes and plays an important role in starch saccharification and the production of various flavor compounds [53,54]. *Lactiplantibacillus plantarum* and other LAB species contribute lactic acid and other organic compound production in Baijiu fermentation, which are necessary for key flavor compounds formation in Baijiu [55,56]. In addition to *Saccharomycopsis fibuligera*, *Lichtheimia* species are also important functional fungi responsible for starch degradation in the early stage of Baijiu fermentation [7,10]. Among the dominant fungal species, *Lichtheimia ramosa* showed a higher relative abundance in QD and in ID (Figure 4b). Co-occurrence networks analysis showed that QD had a more stable microbial community than ID (Figure 4e,f). In general, the QD network had more connections and associated species, longer average path lengths, and a higher clustering coefficient in structure, which may maintain the stability and diversity of the microbial community in QD. The results of this study show that the quality of Daqu is determined by the composition of microbial communities in Daqu.

The fermentation experiment showed that the quality of Daqu significantly affects the microbial communities involved in Baijiu fermentation and consequently the ethanol yield and flavor compound production in the end product. The relative abundances of amylolytic microbes including *Bacillus licheniformis*, *Lichtheimia ramosa*, and *Saccharomycopsis fibuligera* were much higher in the QD than in the ID fermentation at the beginning (Figure 5c,d; Table S4), resulting in much more efficient hydrolyzation and saccharification of starch in the QD fermentation set, as indicated by its remarkably higher moisture content (Figure 6b). Contrarily, the ID fermentation set had a higher relative abundance of *Bacillus subtilis* and *Staphylococcus gallinarum* at the beginning, which are responsible for the ropiness in bread spoilage and the formation of guaiacol (an off-odor compound) in Japanese sake brewing [57,58]. These bacteria probably also negatively affect Baijiu fermentation.

Flavor compounds are important indicators of Baijiu quality. Compared to the ID fermentation set, the QD set exhibited more efficient fermentation, as indicated by more weight loss (Figure 6a), and thus yielded much more glycerol, esters, and ethanol (Figure 6d,e). Glycerol is a common component in fermented food (such as liquor, bread dough, and kefir) and is one of the major byproducts produced from sorghum alcohol fermentation [59], imparting a sweet flavor to Baijiu. A reasonable inference is that QD contained more culturable yeasts (Figure 2), thus producing more glycerol during the fermentation process, and glycerol served as a feedstock for the synthesis of other flavor compounds [60]. Ethyl acetate, ethyl lactate, ethyl caproate, and ethyl butyrate play key roles in determining the flavor of Baijiu [61]. The contents of ethyl acetate, ethyl lactate, and ethyl caproate were higher in the QD than in the ID fermentation set (*p* < 0.05). The ID fermentation set produced more ethyl butyrate (Figure 6d), which can be attributed to *Clostridium tyrobutyricum* (Figure 5b, Table S4). This species has been identified as one of the dominant bacterial species in pit mud and is associated with the production of ethyl butyrate and butanoic acid in strong-flavor Baijiu [62]. Though the content of acetic acid was higher in the QD than in the ID fermentation set, and the content of lactic acid was similar between the two sets, the total titratable acidity was higher in the ID fermentation product (Figure 6c), implying that more of other acids were formed in the latter. The unidentified acids will also have an impact on the yield of ethanol and the flavor of the end product.

The fermentation process of Daqu is actually an enrichment process of the functional microbes that are required for Baijiu fermentation. Indeed, the present study shows that the quality of Daqu depends on its microbial community. The microbial community assembly is sensitive to many different factors, including the source microbes from the raw materials and surrounding environment, temperature, moisture, and ventilation. Old workshops with a long Daqu fermentation history usually produce a higher proportion of high-quality Daqu than new workshops, mainly due to the established and steady microbial community being the source of microbes. Maintenance of even temperature and moisture throughout the workshop is crucial for the production of qualified Daqu and for the quality uniformity of Daqu breaks put in different places in the workshop. However, the temperature and moisture in a workshop for traditional Daqu making are usually artificially controlled through natural ventilation by opening or closing windows. Evidently, temperature, moisture, and source microbe undulations in different parts of the workshop, and especially in different seasons, are unavoidable. Therefore, the quality of Daqu breaks produced by traditional workshops usually varies remarkably. There is an increasing demand for modern Daqu-making workshops equipped with air-conditioning systems which can control the temperature, humidity, and even CO_2_ and O_2_ concentrations accurately throughout the workshop and thus minimize the effects of undulating climate factors on the quality of Daqu. The previous studies on the microbial community in Daqu [29,32], and this present study comparing the microbial communities between QD and ID, will certainly be helpful for the development of synthetic microbial consortia as the inoculating source of functional microbes for Daqu fermentation in accurately air-conditioned workshops. We believe that the quality of Daqu can be accurately controlled using updated technology.

## 5. Conclusions

Daqu is essential for Baijiu production by providing enzymes and functional microbes responsible for starch degradation and saccharification, ethanol fermentation, and flavor compound generation in the fermentation process of Baijiu. The type of Daqu determines the style of Baijiu [5,7,14,52]. The quality of Daqu should certainly affect the quality of Baijiu, but we did not find any studies showing evidence for this. The quality of Daqu produced in different workshops and in different seasons usually varies because Daqu is manually manufactured by spontaneous fermentation in an open environment, and the factors such as temperature and air moisture which affect microbial growth on and in Daqu bricks cannot be accurately controlled. The quality of Daqu is usually assessed based on subjective sensory parameters supplemented with starch saccharification and fermentation ability tests [29,32]. We show here that the quality of Daqu is determined by its microbial community, which in turn affects the microbial community in Baijiu fermentation and the yield and quality of the end product. Microbiological analyses such as counts of total yeasts and of specific functional species such as *Saccharomycopsis fibuligera*, *Lichtheimia ramosa*, and *Bacillus licheniformis* should be considered for a more reliable and objective assessment of Fen Daqu quality. Further microbiome research comparing more samples of Daqu of different quality is needed for establishing a microbiological standard for the quality of Daqu.

## Data Availability

The sequence data obtained in this study have been submitted to the Genome Sequence Archive of the National Genomics Data Center [63], and they have been released to the public with the accession number CRA009281.

## Supplementary Materials

The following supporting information can be downloaded at: https://www.mdpi.com/article/10.3390/foods12152936/s1, Table S1: Analysis of similarities (ANOSIM) test for differences of microbial communities in qualified (QD) and inferior (ID) Daqu and at the beginning (Day 0) and the end (Day 28) of the fermentation tests using QD and ID, respectively; Table S2: Major microbial compositions of qualified (QD) and inferior (ID) Daqu; Table S3: Co-occurrence network properties of qualified (QD) and inferior (ID) Daqu; Table S4: Major microbial community at the beginning and end of fermentation; Figure S1: The production process of Fen Daqu; Figure S2: Illustration of laboratory-scale fermentation; Figure S3: Rarefaction curve analysis of bacteria and fungi; Figure S4: Microbial community compositions and co-occurrence networks in qualified (QD) and inferior (ID) Daqu; Figure S5: Linear discriminant analysis (LDA) effect size (LEfSe) of QD and ID; Figure S6: Linear discriminant analysis (LDA) effect size (LEfSe) tests of the initial (Day 0) and the final (Day 28) microbial communities in the laboratory-scale fermentation tests using the qualified (QD) and inferior (ID) Daqu, respectively.
