# Supplementary material for: Microbial Community Affects Daqu Quality and the Production of Ethanol and Flavor Compounds in Baijiu Fermentation
Source: Foods. 2023 Aug 2;12(15):2936. doi: 10.3390/foods12152936 (PMC10418397; doi:10.3390/foods12152936)
Supplement: Supplementary file 1 [file foods-12-02936-s001.zip › foods-2526680 - supplementary figures - for publish.pdf]

Supplementary figures

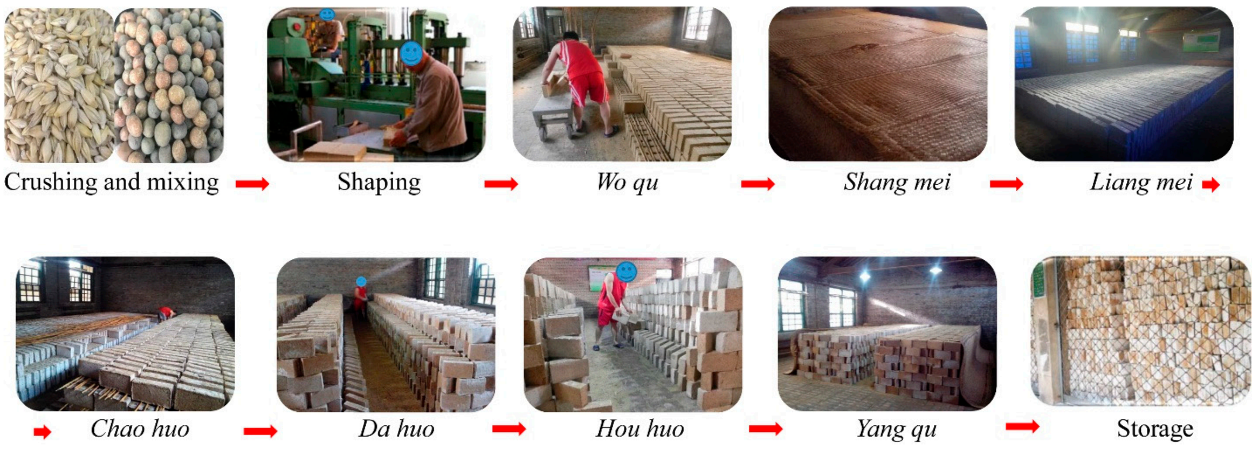

Fig. S1. The production process of Fen Daqu.

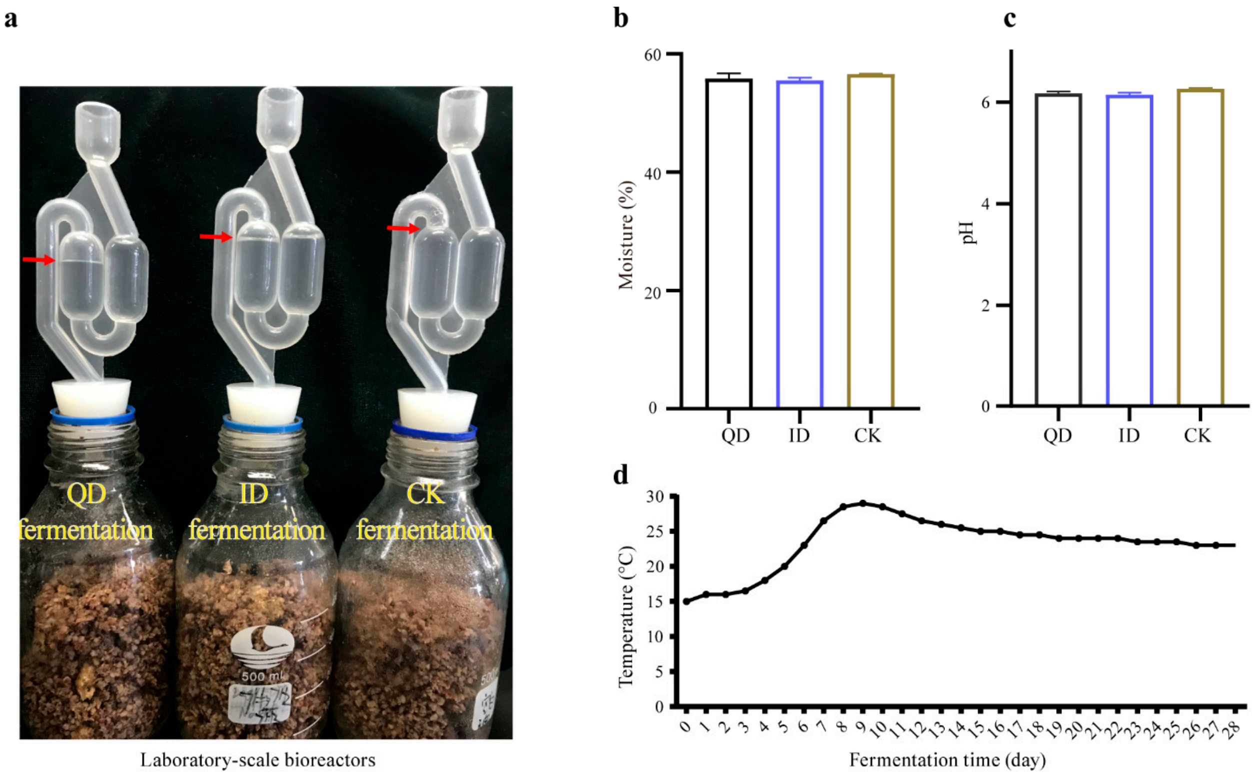

Fig. S2. Illustration of laboratory-scale fermentation. (a) Laboratory-scale bioreactors. The red arrows indicate the change in the water level after seven days of fermentation. (b-c) The initial moisture and pH of the laboratory-scale fermentation sets. (d) The temperature profile of the fermentation. CK, control group.

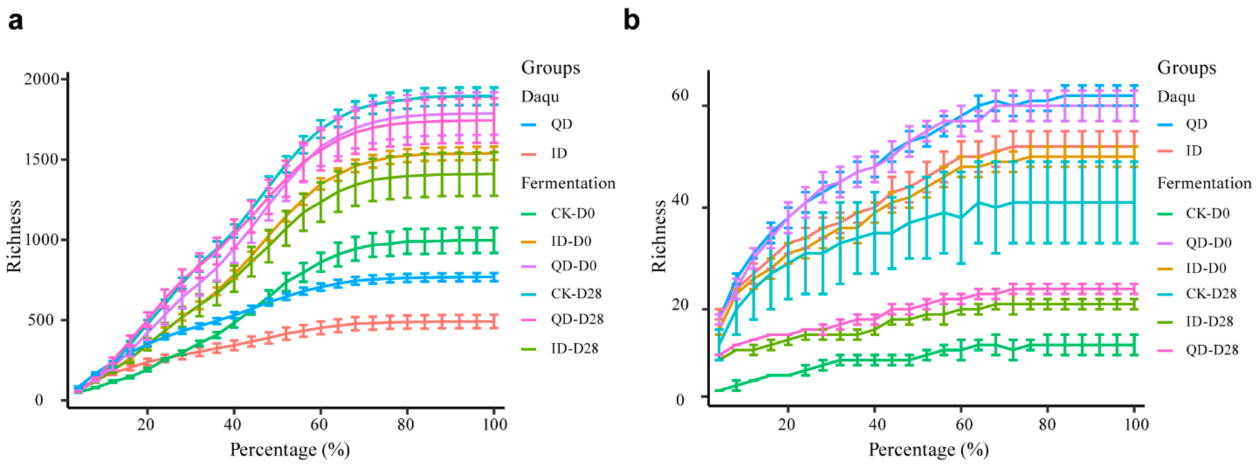

Fig. S3. Rarefaction curve analysis of (a) bacteria and (b) fungi.

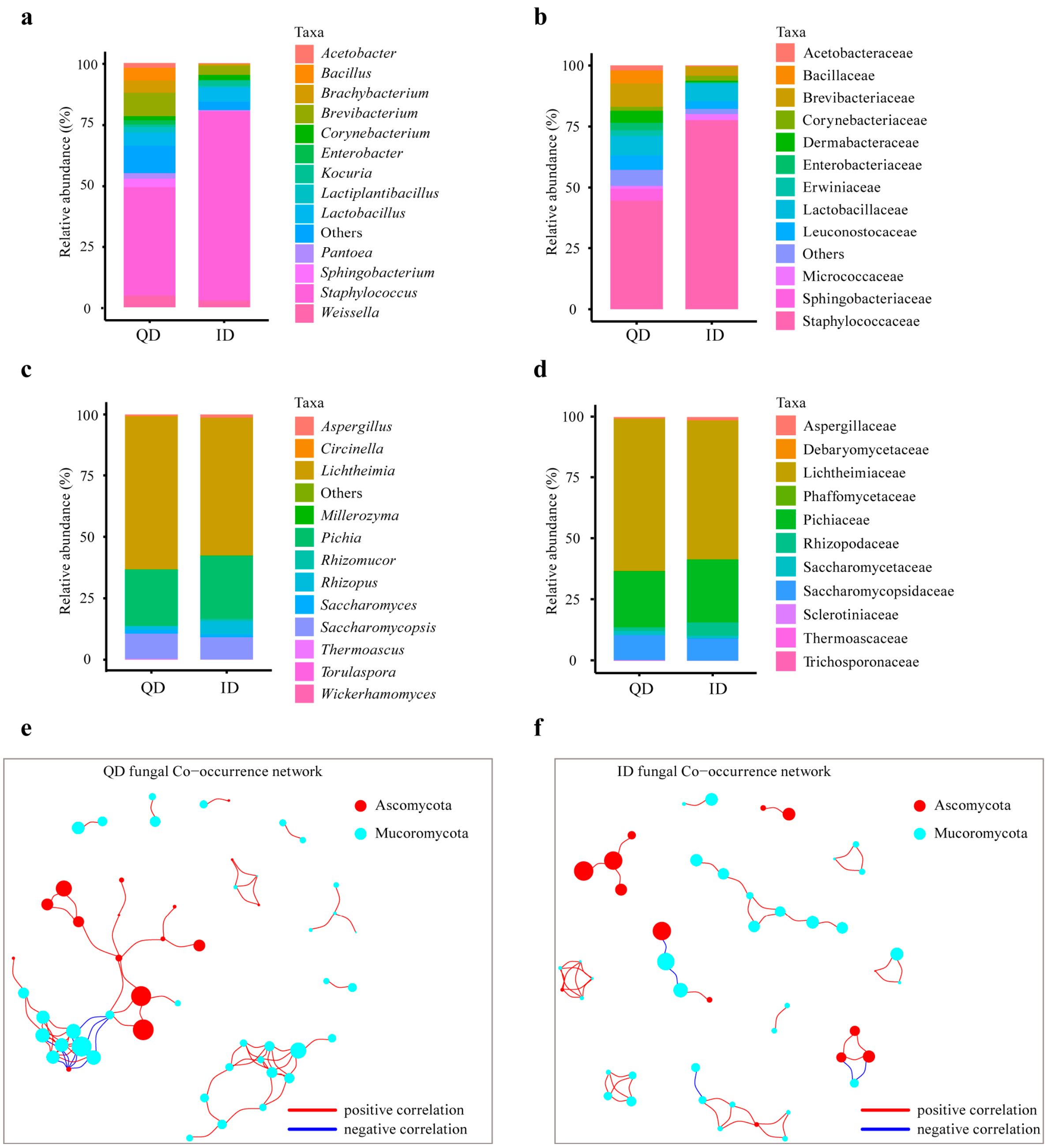

Fig. S4. Microbial community compositions and co-occurrence networks in qualified (QD) and inferior (ID) Daqu. (a) Bacterial and (c) fungal community composition at the genus level. (b) Bacterial and (d) fungal community composition at the family level. Fungal co-occurrence networks of QD (e) and ID (f) were constructed based on ASVs. Nodes represent ASVs and colored by phylum, and links between the nodes show significant correlations.

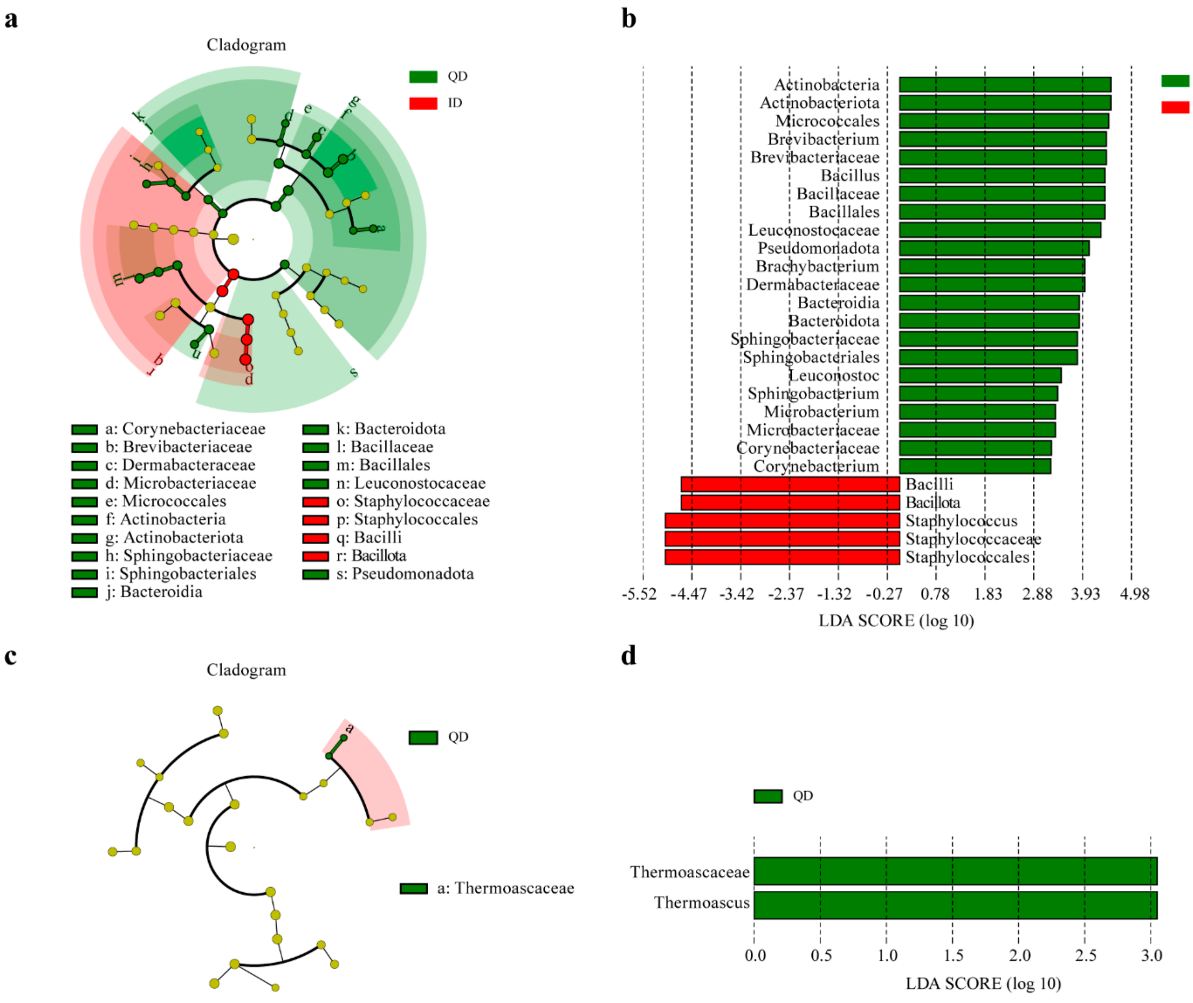

Fig. S5. Linear discriminant analysis (LDA) effect size (LEfSe) of QD and ID (LDA> 3, $P < 0.05$). Cladogram of the bacteria (a) and fungi (c). Significant discriminant taxon nodes of QD and ID are represented by green and red, respectively, while no discriminant taxon nodes are represented by yellow. The LDA score indicates the level of differentiation between QD and ID, and the horizontal bar chart showing discriminant taxa of the bacteria (b) and fungi (d).

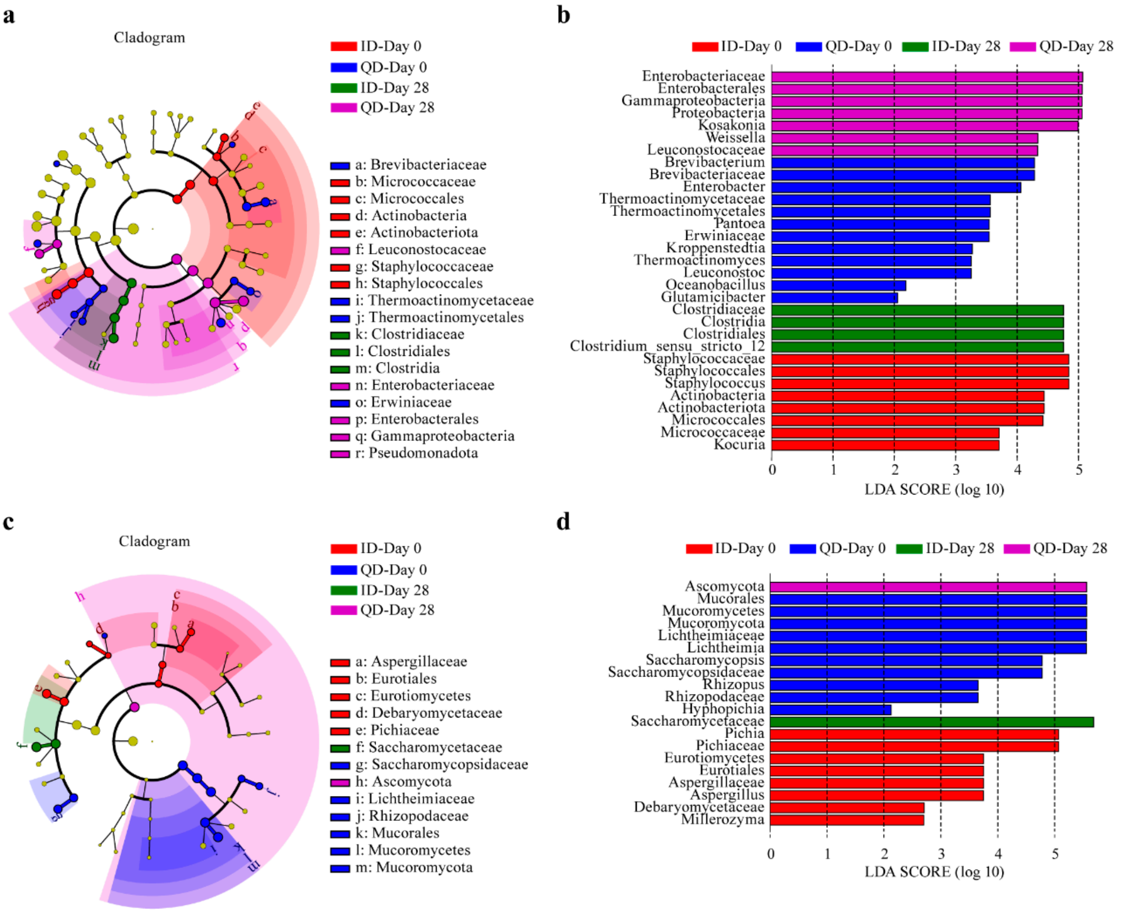

Fig. S6. Linear discriminant analysis (LDA) effect size (LEfSe) tests of the initial (Day 0) and the final (Day 28) microbial communities in the laboratory-scale fermentation tests using the qualified (QD) and inferior (ID) Daqu, respectively (LDA> 2, P <0.05). Cladograms of the bacteria (a) and fungi (c) are shown. Significant discriminant taxon nodes of ID-Day 0, QD-Day 0, ID-Day 28 and QD-Day 28 are shown by red, blue, green and purple, respectively, and non discriminant taxon nodes are represented by yellow. The LDA scores indicate the level of differentiation among ID-Day 0, QD-Day 0, ID-Day 28 and QD-Day 28, and the horizontal bar charts represent discriminant taxa of the bacteria (b) and fungi (d).
